# DNA replication licensing and cell cycle kinetics of oligodendroglial tumours

**DOI:** 10.1038/sj.bjc.6601949

**Published:** 2004-06-15

**Authors:** S B Wharton, S Hibberd, K L Eward, D Crimmins, D A Jellinek, D Levy, K Stoeber, G H Williams

**Affiliations:** 1 Academic Unit of Pathology, University of Sheffield, UK; 2 Department of Histopathology, Royal Hallamshire Hospital, Sheffield, UK; 3 Wolfson Institute for Biomedical Research, University College London, London, UK; 4 Department of Neurosurgery, Royal Hallamshire Hospital, Sheffield, UK; 5 Clinical Oncology, Weston Park Hospital, Sheffield, UK; 6 Department of Histopathology, University College London, London, UK

**Keywords:** Ki67, MCM, Geminin, p21, DNA replication licensing, oligodendroglioma

## Abstract

The convergence point of growth-signalling pathways that control cell proliferation is the initiation of genome replication, the core of which is the assembly of pre-replicative complexes (pre-RCs), resulting in chromatin being ‘licensed’ for DNA replication in the subsequent S phase. The Mcm2–7 complex is a core constituent of the pre-RC, whose recruitment to replication origins is dependent on the Cdt1 loading factor. Geminin is a potent inhibitor of the initiation of DNA replication by preventing Mcm2–7 assembly at origins via its interaction with Cdt1, ensuring genomic integrity through suppression of re-initiation events in S phase. Here we investigate the regulation of Ki67, Mcm2, p21, caspase 3 and Geminin in a series of 55 oligodendrogliomas to provide an integrated picture of how cellular proliferation and programmed cell death are dysregulated in these tumours. Geminin does not behave as an inhibitor of cell proliferation, its labelling index rising with increasing growth fraction as defined by Ki67 or Mcm2 expression. Geminin is expressed in a higher proportion of cells in higher grade tumours (*P*<0.001) and shows a strong correlation to proliferation and replication licensing (*P*<0.01), but not apoptosis. Increasing tumour anaplasia is not associated with loss of Geminin. Importantly, the G1 phase of the proliferative cell cycle, as assessed by the Geminin/Ki67 ratio, shortens with increasing anaplasia, providing new potential algorithms for prognostic assessment. Origin licensing proteins thus provide powerful novel tools for assessment of tumour cell cycle kinetics in routinely processed surgical biopsy material.

The initiation of chromosomal replication is a critical decision point in cell proliferation downstream of cell signalling and DNA transcription. This final and critical step in growth control lies at the convergence point of all oncogenic signalling and transduction pathways that trigger cell proliferation (Williams and Stoeber, 1999; Stoeber *et al*, 2001). Initiation of chromosomal replication is dependent on sequential assembly of multi-protein complexes, referred to as pre-replicative complexes (pre-RCs), at replication origins along the chromosomes during late mitosis and early G1 phase of the cell cycle. Assembly of pre-RCs onto chromatin results in origins being ‘licensed’ for replication during the subsequent S phase (Blow and Hodgson, 2002). The initial step in origin licensing is the binding of the origin recognition complex (ORC) to chromatin. The origin recognition complex functions as a landing platform for two loading factors, cell division cycle (Cdc)6 and Cdt1, which in turn recruit the minichromosome maintenance (MCM) complex comprised of subunits Mcm2–7 to the origin (Lei and Tye, 2001). At the G1 to S phase transition, bi-directional replication forks are established at licensed origins by the concerted action of cyclin-dependent kinases (CDKs) (Nishitani and Lygerou, 2002) and the ASK-dependent Cdc7 kinase (Masai and Arai, 2002). Firing of origins triggers a conformational change in the macromolecular origin-licensing complex, and results in recruitment of the single-strand binding protein RPA and additional initiation/elongation factors to origins. During this process, the DNA helix is unwound by the helicase activity of the MCM complex (Labib and Diffley, 2001), and replication is initiated by the primase activity of DNA polymerase-*α* (Bell and Dutta, 2002).

The initiation of chromosomal replication is tightly coupled to removal of the license and thus prevention of re-licensing following origin firing. This step is critical as replication must occur once, and only once, per cell cycle to ensure genomic stability. As such, human cells have adopted several strategies for preventing origin re-licensing. These include elevated CDK activity during the latter half of the proliferative cycle, resulting in activation and/or removal of replication-licensing factors, changes in gene expression and/or cell-cycle-regulated ubiquitin-mediated proteolysis of replication-licensing factors, and expression of a negative regulator of origin licensing known as Geminin during the S, G2 and M phases (Blow and Hodgson, 2002; Nishitani and Lygerou, 2002). Geminin acts as an inhibitor of DNA replication initiation via its interaction with the loading factor Cdt1 and subsequent inhibition of MCM loading onto chromatin (Wohlschlegel *et al*, 2000; Tada *et al*, 2001).

We have previously shown that repression of origin licensing is a ubiquitous route by which the proliferative capacity of cells is lowered as cells exit from cycle. Withdrawal from cycle into quiescent, differentiated or senescent states is coupled to downregulation of the MCM helicase proteins and the Cdc6 loading factor (Stoeber *et al*, 2001; Blow and Hodgson, 2002). Importantly, we have demonstrated that dysregulation of these replicative factors occurs in dysplastic states, indicating that it is an early event in tumorigenesis (Williams *et al*, 1998; Freeman *et al*, 1999; Stoeber *et al*, 1999, 2002; Going *et al*, 2002). Although Geminin is a potential inhibitor of cell proliferation (McGarry and Kirschner, 1998; Wohlschlegel *et al*, 2000), a regulator of differentiation (Kroll *et al*, 1998), and may be required for maintenance of genomic integrity (Saxena and Dutta, 2003), its role in tumour progression remains to be determined.

Here we investigate the regulation of DNA replication-licensing factors Mcm2 and Geminin in a series of oligodendrogliomas to examine the potential linkages between aberrant Geminin expression and tumour progression. These tumours are relatively homogeneous, facilitating accurate assessment of proliferation indices. Moreover, we have previously demonstrated that expression of the Mcm2 origin-licensing factor rises with increasing tumour grade and other markers of proliferation in this tumour type (Wharton *et al*, 2001). Our study includes a comparison of the regulation of Geminin with the *bona fide* cell cycle inhibitor p21/WAF and an analysis of potential linkages between these cell cycle regulators and apoptosis.

## MATERIALS AND METHODS

### Clinical material

A total of 55 surgical specimens of oligodendroglioma were retrieved from the files of the Histopathology Department of the Royal Hallamshire Hospital for the period 1985–1997. Ethical approval for the study was obtained from the local research ethics committee. Eight of the cases showed focal areas of astrocytic differentiation. In all, 47 of the cases were first diagnoses and eight were recurrent tumours. All of the cases were reviewed by a neuropathologist for confirmation of diagnosis and histological grade according to the World Health Organisation (WHO) criteria (Reifenberger *et al*, 2000a, 2000b). In total, 25 cases were graded as WHO grade II and 30 as anaplastic tumours, WHO grade III. The biopsies were derived from 22 female and 33 male patients. World Health Organisation grade II tumours were from patients with a mean age of 37.4 years (s.d. 15.6), while WHO grade III tumours tended to be from older patients, of mean age 44.6 years (s.d. 14.5).

### Cell culture conditions

MOLT-4 human leukaemic lymphocytes (ATCC CRL-1582; Rockville, MD, USA) were cultured in CO_2_-independent media (Leibovitz (L-15)) supplemented with 50 U ml^−1^ penicillin G, 50 *μ*g ml^−1^ streptomycin sulphate and 10% foetal bovine serum (all from Invitrogen, Paisley, UK) and maintained at 37°C. Using a novel method for cell synchronisation (membrane elution), minimally disturbed early G1-phase cells were collected and followed during synchronous growth as previously described (Thornton *et al*, 2002; Helmstetter *et al*, 2003). Samples from these synchronous batch cultures were removed at specific cell cycle time points for determination of cell concentrations, cell sizes, and analyses of DNA content and protein. Cell density and size were determined using a Coulter Z2 Particle Count and Size Analyzer (Beckman-Coulter, Hialeah, FL, USA). HL-60 cells were obtained from the European Collection of Cell Cultures (ECACC). Asynchronous HL-60 cells were maintained between 1 and 5 × 10^5^ cells ml^−1^ at 37°C in a humidified 5% CO_2_ atmosphere in RPMI 1640 medium supplemented with 2 mM glutamine and 10% FCS.

### Generation of rabbit polyclonal antisera against full-length human Geminin

pET15b-human (*hs*)Geminin (a generous gift from Anindya Dutta; Wohlschlegel *et al*, 2000) was expressed in *Escherichia coli* strain BL21(De3) and purified by Ni-NTA metal affinity chromatography following the manufacturers’ instructions (Qiagen, Crawley, UK). Recombinant *hs*Geminin was further purified using an (HPLC) Hi-load Q sepharose 16/10 column in NaPi buffer and eluted with varying concentrations of 1 M NaCl. Four rabbits were injected with 125 *μ*g of purified *hs*Geminin protein, and received three boost injections over a period of 80 days following a standard protocol (Eurogentech, Seraing, Belgium). The sera were collected and affinity-purified on a CNBr column against 10 mg of recombinant *hs*Geminin protein, eluted with 0.1 M glycine (pH 2.5) and dialysed into PBS, 1% BSA and 0.1% sodium azide. An equal volume of sterile glycerol was added and affinity-purified polyclonal antibodies G92 and G95 were stored at −20°C. Antibody purification was quality controlled by SDS–PAGE for purity and by ELISA. Specificity of the affinity-purified antibodies was demonstrated by immunoblotting of cell lysates and by quenching all immunohistochemical staining after incubating antibodies diluted at working concentrations with equal or less than molar amount of recombinant *hs*Geminin protein for 1 h prior to a standard immunohistochemical staining protocol.

### Flow cytometric analysis of DNA content, Ki67 and Geminin

For cell cycle analysis of DNA content, cell samples were fixed in 80% ethanol and stained with propidium iodide as previously described (Helmstetter *et al*, 2003) before flow cytometric analysis. For determination of Ki67 and Geminin content, cells were fixed as described above, and permeabilised with nonionic detergent using a method modified from Gong *et al* (1995). After centrifugation, cell pellets were resuspended in D-PBS, and either 10 *μ*l of FITC-conjugated Ki67 monoclonal antibody (clone B56) or the matched nonspecific staining control (FITC-conjugated IgG; each from BD PharMingen™, Oxford, UK), or 0.1 *μ*g of affinity-purified rabbit polyclonal anti-Geminin antibody G95, or G95 and recombinant *hs*Geminin protein (1 : 10 ratio). After 2 h incubation at 4°C, cells were washed with D-PBS containing 1% BSA, concentrated via centrifugation and stained with propidium iodide. Analyses of light-scatter properties and DNA/protein content were performed using a FACSCalibur flow cytometer (BD Biosciences, San Jose, CA, USA). Cell doublets were excluded by gating on a dot plot of the width *vs* the area of DNA fluorescence intensity (Erlanson and Landberg, 1998). In most samples, 10^4^ cells were examined and data were stored and analysed using CellQuest™ software (BD Biosciences), Cylchred (V.1.0.0.1) and WinMDI (V.2.7).

### Preparation of protein extracts and immunoblotting

Whole-cell lysates (2 × 10^6^ cells) were prepared for immunoblotting from asynchronous or synchronous batch cultures using a method modified from Harlow and Lane (1999). Cell pellets were resuspended by vigorous vortexing in sample buffer containing 3% SDS, 100 mM DTT, 60 mM Tris (pH 6.8), 0.01% bromophenol blue and 10% glycerol. Equal loading of MOLT-4 (or HL-60) cell lysates was achieved by using the extract from equivalent cell numbers in each lane. Immunoblots were performed as previously described (Stoeber *et al*, 2001) using affinity-purified rabbit polyclonal anti-Geminin antibodies, and antibodies obtained from commercial sources against the following antigens: Mcm2 (clone 46) and PCNA (Clone 24; BD Transduction Laboratories™, Lexington, KY, USA), Cyclin A (C-19; Santa Cruz; CA, USA). Total cell extracts from equivalent cell numbers were separated on 10% SDS–PAGE gels (Invitrogen), before transfer to Hybond-P membranes (Amersham, Little Chalfont, UK), incubation with primary and secondary antibodies (HRP-conjugated anti-mouse or anti-rabbit antibodies (DAKO, Glostrup, Denmark)), and visualisation of immune complexes using enhanced chemiluminescence (Amersham).

### Immunohistochemistry and expression profile analysis

All available slides from each case were reviewed and a block was selected containing a representative area of oligodendroglioma. Immunohistochemistry was performed using antibodies against Mcm2, Ki67, activated caspase 3 and p21, as shown in Table 1

**Table 1:** Conditions for immunohistochemistry

**Antibody**	**Source**	**Dilution**	**Incubation**	**Ag retrieval**
Ki67	Novocastra	1 : 25	1 h RT	MW 20 min Na citrate pH 6
p21	DAKO	1 : 25	1 h RT	MW 20 min Na citrate pH 6
Caspase 3	R&D systems	1 : 600	O/N 4°C	MW 20 min Na citrate pH 6
Mcm2	Transduction laboratories	1:1000	1 h RT	PC 2 min Na citrate pH 6
G92	See text			
G95	See text			

RT=room temperature, O/N=overnight, MW=microwave, PC=pressure cooker.

. Apoptotic bodies were assessed on H&E-stained sections. Immunohistochemistry was performed using an ABC method and the signal visualised using diaminobenzidine. Serial sections were cut at 4 *μ*m onto APES-coated slides, or at 3 *μ*m onto ChemMate slides (DAKO) for Ki67, Mcm2 and Geminin preparations. Areas of highest cellularity and expression were used for quantitation, as previously described (Wharton *et al*, 1998). For each marker, a percentage labelling index was obtained from a count of at least 1000 cells using an eyepiece graticule.

### Statistical analysis

Statistical analyses were performed using the Statistical Package for the Social Science (SPSS, V.10.1). Labelling indices between grades were compared using the Mann–Whitney *U*-test and correlation determined using Pearson's coefficient of correlation.

## RESULTS

### Generation and characterisation of rabbit polyclonal antibodies against human Geminin

**Figure 1:**
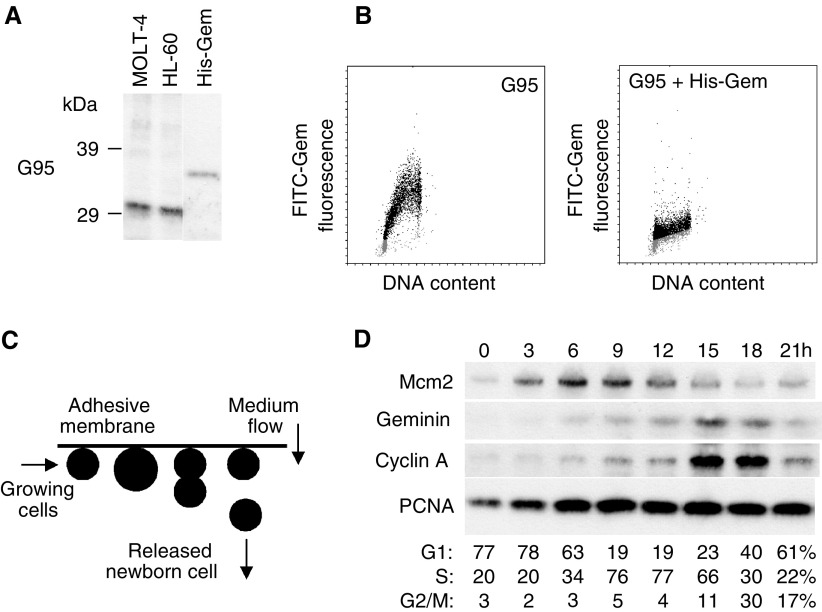
(**A**) Immunoblots of total cell lysates prepared from asynchronously proliferating MOLT-4 and HL-60 cells and rec. human Geminin with anti-Geminin antibody G95. Rabbit polyclonal affinity-purified antibody G95 detected a single protein with a molecular mass of ∼33 kDa in total lysates from MOLT-4 (lane 1) and HL-60 (lane 2) cells, and recognised nanogram quantities of rec. Geminin (lane 3; ∼34 kDa). (**B**) Bivariate flow cytometry of asynchronous MOLT-4 cells with G95 alone, or G95 plus rec. Geminin, further supports the notion that the antibody is specific for human Geminin. Note that cells in G1 (2C; grey dots) are Geminin-negative, whereas cells in S–G2–M (black dots) are positive for Geminin. Pre-incubation of G95 with rec. Geminin results in loss of cell cycle periodicity and little Geminin expression is detected at any stage of the cell cycle. (**C**) Schematic of membrane elution. Asynchronously growing MOLT-4 cell cultures are immobilised on surfaces such that gravity coupled with cell division results in release of one daughter cell, while the other remains surface-bound. Newborn early G1-phase cells are continuously released in the effluent and grow synchronously without evidence of disturbance. (**D**) Immunoblots of origin-licensing factors and control proteins in synchronously proliferating MOLT-4 cells. Protein levels of Mcm2, Geminin, Cyclin A and PCNA were determined in lysates from equivalent numbers of cells isolated from synchronous batch cultures at the indicated times.

We raised rabbit polyclonal antisera G92 and G95 against bacterially expressed full-length human Geminin. In immunoblots of total cell lysates from asynchronously proliferating human MOLT-4 leukaemic lymphocytes (Figure 1A

, lane 1) and human promyelocytic HL-60 cells (Figure 1A, lane 2), affinity-purified antibody G95 detected a single protein with an estimated molecular mass of ∼33 kDa, consistent with the reported electrophoretic mobility of human Geminin (McGarry and Kirschner, 1998; Wohlschlegel *et al*, 2000), while the pre-immune sera did not (data not shown). Antibody G95 also recognised recombinant His_6_-tagged Geminin with a molecular mass of ∼34 kDa (Figure 1A, lane 3). In addition, bivariate flow cytometric analyses of Geminin expression and DNA content in asynchronously proliferating MOLT-4 cells showed that Geminin was absent in cells (represented as dots) with a 2C (or G1 phase) DNA content (grey dots) and increased during S and G2 phases of the cell cycle (black dots; Figure 1B), consistent with previous studies in HeLa cells (McGarry and Kirschner, 1998; Wohlschlegel *et al*, 2000). Pre-incubation of antibody G95 with a 1 : 10 ratio of recombinant Geminin resulted in loss of detection of this cell cycle-specific expression (Figure 1B). Affinity-purified antibody G92 also recognised the ∼33 kDa band in total cell lysates, but showed weak crossreactivity with nonspecific bands of higher molecular mass (data not shown). Antibody G95 was therefore chosen for subsequent detailed immunoblot analysis of Geminin expression during the proliferative cell cycle.

### Cell cycle expression of origin-licensing factors and tumour cell kinetics

Determination of the molecular and temporal events comprising the process of mammalian DNA replication often requires the use of synchronous cell populations. We have examined expression of the standard proliferation marker Ki67, the DNA replication-licensing factor Mcm2 and the repressor of origin-licensing Geminin during the proliferative cell cycle, using a novel, nonchemical method for cell synchronisation (membrane elution; Thornton *et al*, 2002; Helmstetter *et al*, 2003; Figure 1C). We have employed membrane elution because use of chemical methods for cell synchronisation may account in part for reported discrepancies in temporal periodicities and subcellular localisation of replication proteins such as Orc1 (Pak *et al*, 1997; Kreitz *et al*, 2001; Okuno *et al*, 2001; Li and DePamphilis, 2002) and Cdc6 (Fujita *et al*, 1999; Jiang *et al*, 1999; Mendez and Stillman, 2000; Petersen *et al*, 2000).

Newborn (early G1-phase) MOLT-4 cells isolated by membrane elution progressed through a full cycle of synchronous growth, as demonstrated by FACS analysis of the DNA content (Figure 1D). In total cell extracts prepared from these synchronous batch cultures, protein levels of the DNA replication-licensing factor Mcm2 increase linearly in early G1 phase up to a maximum in S phase before decreasing during the latter part of the cell cycle (Figure 1D). Moreover, cell synchronisation by membrane elution has revealed a similar cell cycle-dependent periodicity in Mcm5 and Mcm7 expression (Eward *et al*, manuscript submitted), findings that may have been previously obscured by metabolic perturbation due to the use of chemical synchronisation agents. Previous work has suggested that Geminin is present during S, G2 and early mitosis of the proliferative cell cycle before ubiquitination by the anaphase-promoting complex (McGarry and Kirschner, 1998; Wohlschlegel *et al*, 2000; Nishitani *et al*, 2001; Tada *et al*, 2001). Using our newly generated affinity-purified polyclonal anti-Geminin antibody G95 (Figure 1A), we found little to no expression of this repressor of origin licensing during G1 phase, with detectable levels at the G1/S transition and increasing linearly during the remainder of the cycle before mitotic degradation (Figure 1D). Notably, the Geminin expression profile is similar to Cyclin A, another oscillating cell cycle regulator which, like Geminin, is degraded at the metaphase to anaphase transition via the ubiquitin–proteasome pathway (Figure 1D) (Yam *et al*, 2002). In contrast to the proteins described above, protein levels of proliferating cell nuclear antigen (PCNA) remained constant during synchronous progression through the cell cycle (Figure 1D). Moreover, the fraction of MOLT-4 cells expressing the standard proliferation marker Ki67 during synchronous growth (85–95%; determined by FACS analysis) remained constant and was similar to the proportion that was Ki67-positive in asynchronous culture (data not shown). Geminin therefore provides a precise estimate of the S-G2-M growth fraction in dynamic cell populations and the ratio of Geminin/Ki67 provides information about the relative length of the G1 phase of the proliferative cell cycle (Figure 1D).

### Correlation of Geminin expression to replication licensing and cell proliferation

Ki67 and Mcm2 both demonstrated an increased labelling index in WHO grade III compared to grade II tumours (*P*<0.01), reflecting greater proliferation with increasing anaplasia (Table 2

**Table 2:** Labelling indices – descriptive statistics

**Grade**	**Statistic**	**Ki67**	**Mcm2**	**AI**	**Csp3**	**p21**	**G92**	**G95**
II	Mean (s.d.)	11.4 (8.5)	16.0 (15.3)	0.8 (0.5)	1.8 (2.5)	2.3 (2.7)	2.5 (1.8)	2.4 (1.9)
								
	Median (IQR)	8.8 (11.7)	10.2 (16.8)	0.6 (0.8)	0.9 (1.6)	1.5 (3.4)	2.5 (2.5)	2.3 (2.8)
								
III	Mean (s.d.)	22.1 (16.3)	40.0 (25.1)	1.1 (0.7)	2.4 (2.5)	4.4 (5.4)	8.9 (9.8)	8.5 (6.9)
								
	Median (IQR)	21.3 (18.2)	37.8 (44.6)	0.9 (0.8)	1.6 (3.2)	3.2 (4.5)	6.4 (5.1)	6.4 (7.2)
								
	*P*-value^*^	**0.002**	**<0.001**	0.058	0.335	0.061	**<0.001**	**<0.001**

AI=apoptosis index based on H&E counts, IQR=interquartile range, *P*-value^*^=for grade III *vs* grade II tumours, Mann–Whitney *U*-test.

). Values for Mcm2 obtained in this study showed a pattern of increasing expression with grade similar to that observed previously by us in a separate series of oligodendroglial tumours (Wharton *et al*, 2001).

**Figure 2:**
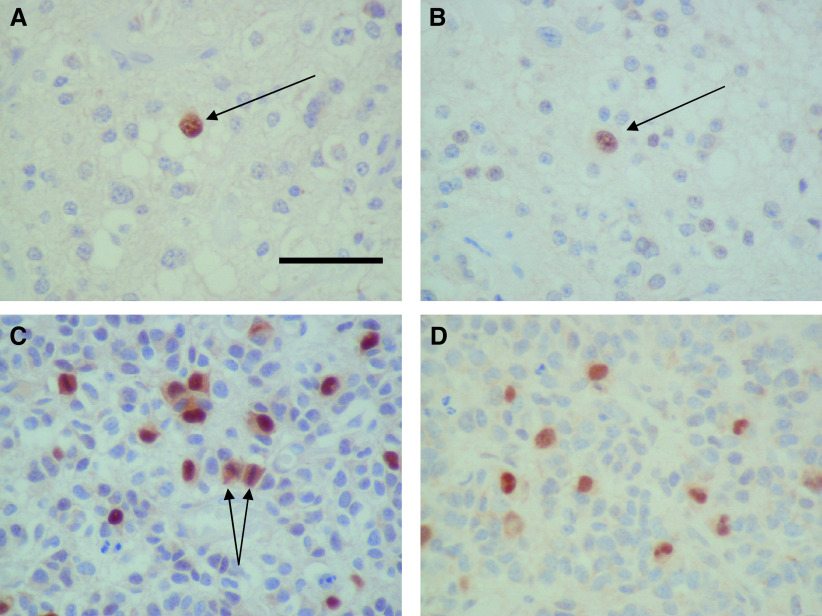
Geminin immunohistochemistry. (**A**) Low-grade oligodendroglioma showing a single Geminin-positive nucleus (arrow) stained with G95. (**B**) G92 showing a similar nuclear pattern of staining. (**C**) Anaplastic oligodendroglioma showing multiple nuclei stained with G95. Diffuse cell staining is observed in mitotic figures (arrows). (**D**) Anaplastic oligodendroglioma stained with G92.

**Figure 3:**
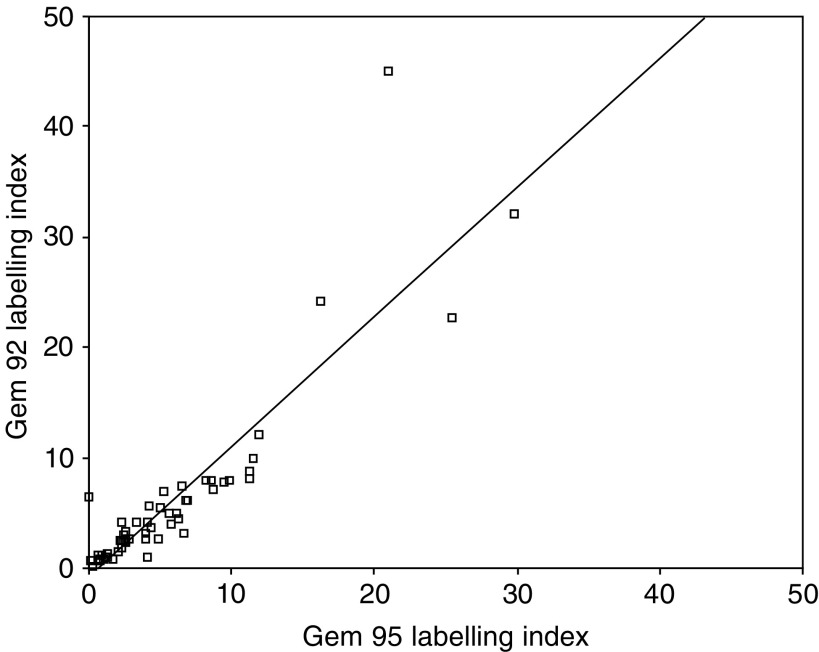
Scatter plot showing a close correlation between labelling indices obtained with G92 and G95.

Both Geminin antibodies, G92 and G95, gave strong, predominantly nuclear staining (Figure 2

). Cytoplasmic staining was noted in some cases, especially with antibody G92. Quantitation of staining with the two antibodies produced comparable results with very good correlation (*r*=0.89, *P*<0.01) (Figure 3

). Although comparable, staining with G95 was stronger and the values for this antibody have been used for subsequent analyses.

**Figure 4:**
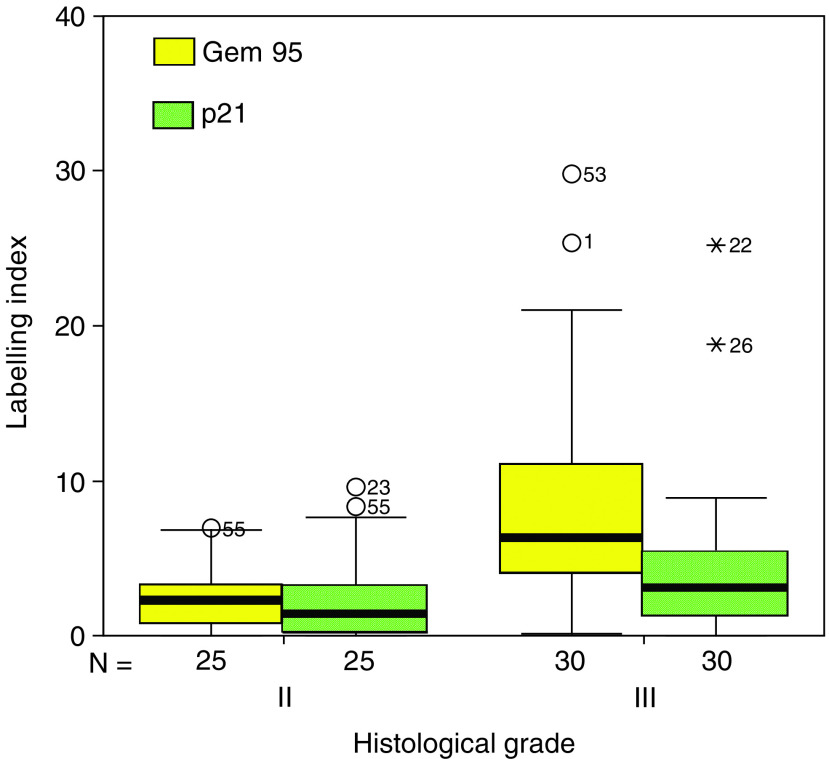
Box-whisker plot showing labelling indices for G95 and p21 in relation to histological grade. Median values are shown as heavy lines within the boxes. Outlying values (>1.5 box lengths from the upper end of the box) are represented as stars or circles.

**Figure 5:**
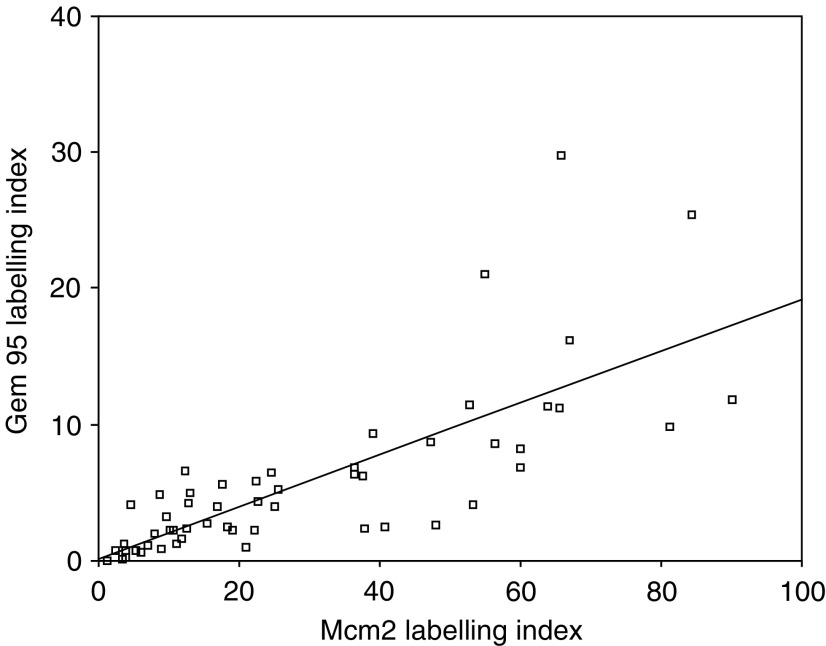
Scatter plot showing a strong linear correlation between Geminin and Mcm2 expression.

Geminin expression showed a clear relationship to grade, with greater labelling indices for both antibodies for grade III *vs* grade II tumours (*P*<0.001), and with a greater variation for the higher grade tumours (Figure 4

, Table 2). Geminin expression showed a close relationship to licensing for proliferation, with a strong quantitative relationship to Mcm2 labelling index (*r*=0.76, *P*<0.01) (Figure 5

). Examination of the scatter plot did not suggest the presence of any distinct clustered subgroup in which the relationship of Geminin expression to chromosome licensing was differently defined.

### Relationship of Geminin to p21/WAF1 expression

**Figure 6:**
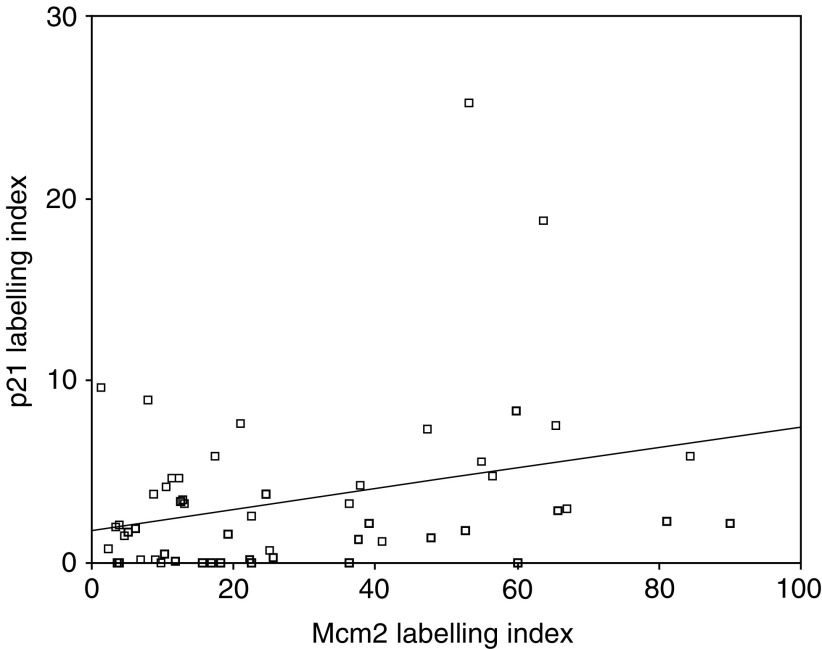
Scatter plot showing a poor relationship between p21 and Mcm2 labelling indices.

We sought to determine whether Geminin behaved similarly to the cell cycle inhibitor p21. Although the p21 labelling index showed a trend towards increased labelling with higher grade, this was not significant. Similarly, although p21 showed a correlation with the Mcm2 proliferation marker, this was very weak (*r*=0.3, *P*<0.05) (Figure 6

). There was no correlation between p21 and Geminin (*r*=0.17, *P*=0.224).

**Figure 7:**
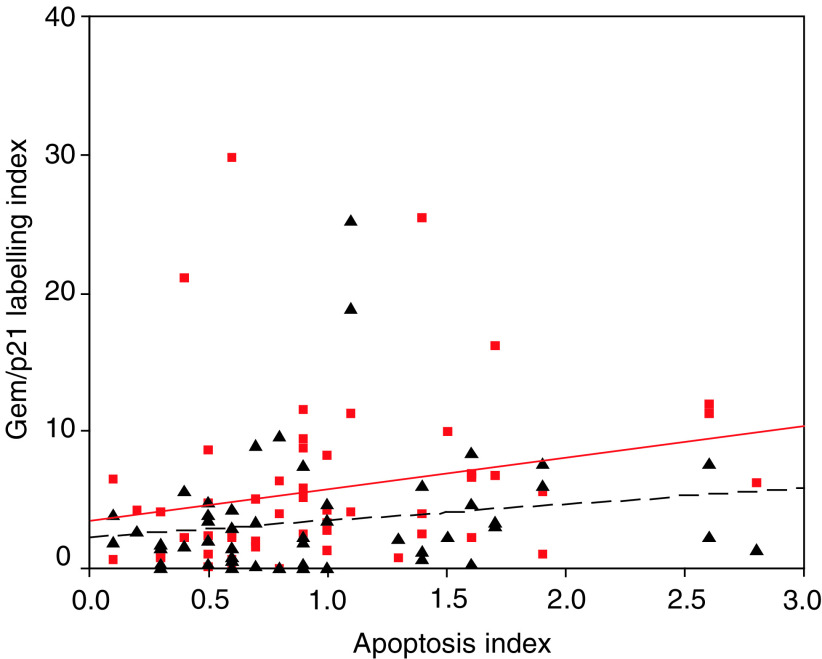
Scatter plot showing the relationship of apoptosis index with G95 (squares and solid line) and with p21 (triangle and dashed line).

Induction of p21 may occur due to activation of p53 and may therefore relate to increasing cellular stress. We therefore sought to determine whether p21 or Geminin expression might correlate with degree of apoptosis. Apoptosis was assayed by means of counts of apoptotic bodies and labelling index for activated caspase 3. Both of these markers demonstrated a nonsignificant trend towards higher labelling with grade. Neither p21 nor Geminin expression demonstrated a significant correlation to levels of apoptosis (Figure 7

).

### The Geminin/Ki67 ratio is a new parameter for determining the relative length of G1 phase in dynamic cell populations

**Figure 8:**
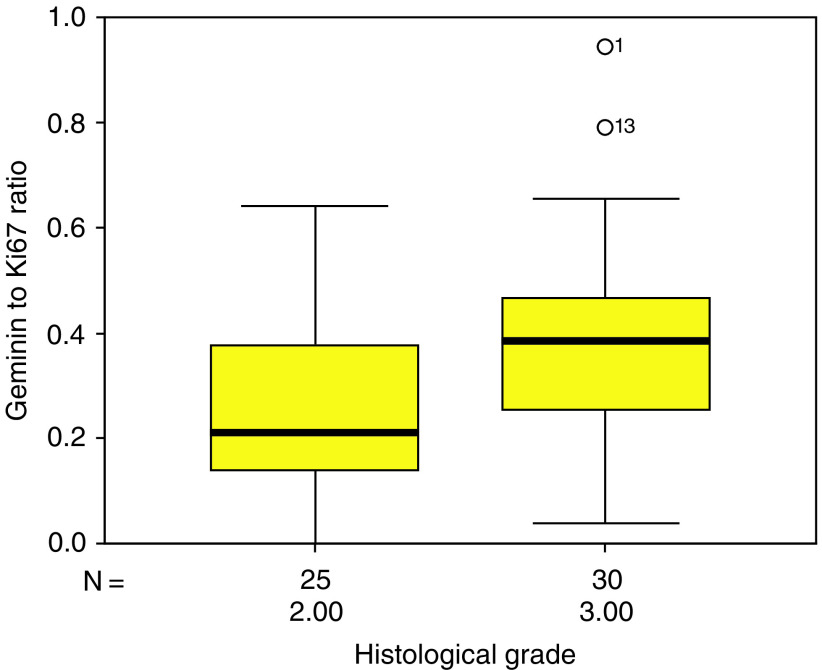
Box plot showing Geminin to Ki67 ratio for grade II and grade III oligodendrogliomas.

A comparison of Geminin/Ki67 ratios between low- and high-grade tumours reveals significant differences. The ratio is significantly higher for the grade III tumours (mean 0.39, s.d. 0.2) than grade II tumours (mean 0.25, s.d. 0.17; *P*=0.005) (Figure 8

), indicating a relatively shorter G1 phase in these tumours. There was no significant correlation between Geminin/Ki67 ratio and apoptosis.

## DISCUSSION

We have used two polyclonal affinity-purified antibodies to detect the expression of Geminin in a series of human oligodendrogliomas. Both antibodies produced very similar quantitative results, and demonstrated a predominantly nuclear pattern of expression, as previously reported in proliferating cell populations (Wohlschlegel *et al*, 2002). Cytoplasmic expression was noted in some cases, particularly using the G92 antibody. The labelling index for Geminin was higher for grade III than for grade II tumours, and showed a strong positive correlation with markers of proliferation (Ki67) and DNA replication licensing (Mcm2). Our data show that expression of this origin licensing repressor is tightly coupled to proliferation in these tumours and do not support the alternative hypothesis that Geminin is an inhibitor of cell proliferation. The loss of Geminin therefore does not appear to be rate-limiting in the establishment of high-grade anaplastic tumours and may indicate that re-licensing of DNA replication does not contribute to genetic instability in these tumours. A similar pattern of expression, whereby Geminin appears to be expressed in proliferating cells, in a manner analogous to cell-cycle progression factors, has been noted in proliferating normal tissues (Wohlschlegel *et al*, 2002).

Oligodendrogliomas may be subdivided into two molecular types, according to whether there is deletion from chromosome 1p and 19q. The presence of 1p/19q loss predicts a longer progression-free survival and a better response to combination chemotherapy (Cairncross *et al*, 1998; Smith *et al*, 2000; Ino *et al*, 2001). Similar cytogenetic abnormalities are observed in tumours which also have areas of astrocytic differentiation (Kraus *et al*, 1995; Maintz *et al*, 1997). These molecular subgroups appear histologically similar (Sasaki *et al*, 2002). It is conceivable, however, that such subgroups differ in their patterns of cell cycle dysregulation. Inspection of scatter plots for our data does not reveal any distinct tumour subgroups in which the relationship of Geminin to that of proliferation licensing might be differently defined, but this is a hypothesis that we are exploring in further work.

We also sought to determine in this tumour type whether Geminin behaved in a similar way to a known cell-cycle inhibitor, p21. p21 (WAF1/CIP1) and p27 bind to, and inhibit, a range of Cyclin/CDK complexes, resulting in inhibition of cell cycle progression (Coqueret, 2003). Previous studies have demonstrated p21 expression in oligodendrogliomas. Some studies have suggested that p21 expression increases with increased tumour grade, with lack of p21 expression associated with a better prognosis (Miettinen *et al*, 2001), although other studies have found little difference in expression between low- and high-grade tumours (Korshunov and Golanov, 2001). In our series, we have shown a nonsignificant trend towards higher p21 labelling indices with higher tumour grade. In contrast, Geminin expression is strongly related to higher tumour grade and growth fraction. This suggests that Geminin behaves somewhat differently from classical cell cycle inhibitors. Data from normal tissue suggest that, whereas Geminin is particularly associated with proliferating cells, p21 is more typically seen in quiescence (Wohlschlegel *et al*, 2002). Similarly, in our study, the lower expression of Geminin in low-grade tumours suggests that this origin-licensing repressor is not required for maintaining tumour cells in quiescence.

Induction of apoptosis in tumours may also reflect cell stress and in oligodendrogliomas, as in many other tumour types, there is a trend towards greater levels of apoptosis in higher grade tumours (Schiffer *et al*, 1997; Wharton *et al*, 1998). Activation of cell cycle inhibitors in higher grade tumours may be a response to greater cell stress. Expression of p21 is induced by p53 (Prives and Hall, 1999), and it is possible that such a pathway might operate in high-grade oligodendrogliomas (Miettinen *et al*, 2001). We therefore hypothesised that there may be a potential linkage between expression of cell cycle inhibitors and apoptosis. We estimated apoptosis using counts of apoptotic bodies in H&E preparations, which correlates well with the TUNEL method (Wharton *et al*, 1998), and by immunohistochemistry to activated caspase 3. Activated caspase 3 has been used as a marker for apoptosis, with a good correlation with other apoptotic indices (Duan *et al*, 2003). In this series, neither p21 nor Geminin showed a statistical relationship to these markers of apoptosis.

The differential expression patterns of Ki67, Mcm2–7 and Geminin during the mitotic cell cycle and their tight downregulation in out-of-cycle states (Stoeber *et al*, 2001; Wohlschlegel *et al*, 2002) provide a novel and powerful multi-parameter analysis for assessment of *growth fraction* and *cell cycle kinetics* in human tissues and tumours. Using membrane elution, we have shown that Geminin expression is restricted to the S–G2–M phase of the proliferative cell cycle in human cells (Figure 1D). Previous assessment of the S-phase fraction has relied on methodologies such as flow cytometry, *in vitro* incorporation of ^3^H-thymidine (Quinn and Wright, 1990) or *in vitro* DNA synthesis (Mills *et al*, 2000), which are relatively complex, requiring fresh tissue. Here we have demonstrated that the S–G2–M fraction can now be easily assessed in glial tumour specimens simply through application of anti-Geminin antibodies to archival specimens. The Ki67 labelling index identifies all phases of the proliferative cell cycle (G1, S, G2, M), and the Geminin labelling index identifies the fraction of cells in S–G2–M. The Geminin/Ki67 ratio is therefore an indicator of the relative length of the G1 phase. Proliferating cells with a short G1 phase will approximate to a Geminin/Ki67 ratio of ∼1, whereas cells with a prolonged G1 phase will approximate to a Geminin/Ki67 ratio of ∼0. Interestingly, significant differences are observed in the Geminin/Ki67 ratio between grade II and grade III oligodendrogliomas, indicative of an accelerated G1 phase in anaplastic tumours. This novel approach thus allows a detailed assessment of tumour cell cycle kinetics that can be routinely applied to archival specimens. We and others have already demonstrated that the MCM replication-licensing factors can be exploited as prognostic markers in cancer diagnosis (Meng *et al*, 2001; Ramnath *et al*, 2001; Wharton *et al*, 2001; Gonzalez *et al*, 2003; Kruger *et al*, 2003). Analysis of a range of different tumour types including glial tumours is now in progress to determine whether the Geminin origin-licensing repressor and/or the Geminin/Ki67 ratio can be exploited as independent predictors of disease-free survival.

In summary, we have shown that Geminin expression correlates most strongly with proliferation in oligodendrogliomas and not with expression of the p21/WAF1 cell cycle inhibitor. Thus, despite being a potent inhibitor of DNA synthesis, Geminin does not appear to exert an antiproliferative effect in this tumour type. Our studies of the DNA replication-licensing pathway have revealed that high-grade tumours are associated with an accelerated G1 phase. Analysis of constituents of the DNA-licensing pathway in archival material thus provides novel tools for assessment of proliferative capacity and potential new algorithms for improved treatment decisions and prognostication in the management of oligodendroglial tumours.
